# Transcriptomic and Metabolomic Analysis of the Uterine Tissue of Yaoshan Chicken and Its Crossbreeds to Reveal the Molecular Mechanism Influencing Eggshell Quality

**DOI:** 10.3390/genes16040383

**Published:** 2025-03-27

**Authors:** Xiaomeng Miao, Jia Liu, Qian Gong, Fugui Li, Yalan Zhang, Qiyue Liang, Diyan Li, Zhonghua Ning

**Affiliations:** 1 National Engineering Laboratory for Animal Breeding, College of Animal Science and Technology, China Agricultural University, Beijing 100193, China; mxm19920404@126.com (X.M.); m15901003721@163.com (Y.Z.); 18085884126@163.com (Q.L.); 2 Institute of Animal Husbandry and Veterinary Medicine, Guizhou Academy of Agricultural Sciences, Guiyang 550005, China; 3 Guizhou Province Livestock and Poultry Genetic Resources Management Station, Guizhou Provincial Department of Agriculture and Rural Affairs, Guiyang 550001, China; liujia994@126.com (J.L.); 18984162473@163.com (Q.G.); 4 Xinjiang Production & Construction Corps Key Laboratory of Protection and Utilization of Biological Resources in Tarim Basin, College of Life Science and Technology, Tarim University, Alar 843300, China; lifuguiyn@163.com; 5 School of Pharmacy, Chengdu University, Chengdu 610106, China

**Keywords:** eggshell quality, uterus, chicken, transcriptome, metabolome

## Abstract

**Background/Objectives:** Eggshell quality is a critical factor influencing consumer preference and the economic benefits of poultry enterprises, and the uterus is the key site for eggshell synthesis. Yaoshan chicken (YS), an indigenous chicken breed in China, is renowned for its flavorful meat and high-quality eggs. However, its egg production is lower compared to specialized strains. Therefore, the GYR crossbreed was developed by three-line hybridization for YS chicken, which can produce green-shelled eggs with better eggshell thickness and strength than YS chicken (*p* < 0.01). To explore the molecular mechanisms underlying the differences in eggshell quality between GYR and YS chickens, we conducted an integrated transcriptomic and metabolomic analysis. **Methods:** Twelve uterus samples (six from GYR and six from YS chickens) were collected during the period of eggshell calcification at 260 days of age. RNA sequencing (RNA-seq) and liquid chromatography–mass spectrometry (LC-MS/MS) were performed to identify differentially expressed genes (DEGs) and differential metabolites (DMs), respectively. **Results:** A total of 877 DEGs were identified in the GYR group, including 196 upregulated and 681 downregulated genes (|log_2_ (fold change)| > 1, *p*-value < 0.05). Additionally, 79 DMs were detected, comprising 50 upregulated and 29 downregulated metabolites (|log₂ (fold change)| > 1, VIP > 1). Notably, the key DEGs (*SLCO1B3*, *SLCO1B1*, *PTGR1*, *LGR6*, *MELTF*, *CRISP2*, *GVINP1*, and *OVSTL*), important DMs (prostaglandin-related DMs and biliverdin) and signaling pathways (calcium signaling, neuroactive ligand–receptor interaction, arachidonic acid metabolism, bile secretion, and primary bile acid biosynthesis) were major regulators of the eggshell quality. Furthermore, an integrated transcriptomic and metabolomic analysis revealed two significant gene–metabolite pairs associated with eggshell quality: *PTGDS–*prostaglandin E2 and *PTGS1–*prostaglandin E2. **Conclusions:** This study provides a theoretical foundation for the improved eggshell quality of Yaoshan chicken.

## 1. Introduction

The nutrients of eggs are abundant, balanced and cheaper than other animal products, making them a primary source of high-quality protein for human beings and a significant part of food consumption for Chinese residents [1,2]. Egg production is the most important economic index and selection criterion for poultry reproductive performance, as it is closely related to the profitability of poultry enterprises [3]. Previous studies have explored the molecular mechanisms underlying egg-laying performance in poultry and found numerous genes associated with this trait [4,5,6,7]. However, improving the quality of eggshells has also been a positive direction in improving the economic benefit of poultry industries when the genetic progress on egg production is slow.

The eggshell is the natural protective barrier for the contents of eggs, with its strength and thickness playing a crucial role in protecting eggs from the invasion of pathogenic microorganisms and damage during circulation [8]. In addition, eggshell color and the uniformity of pigmentation are among the most intuitive factors manipulating the purchasing decisions of consumers [9]. Green-shelled eggs, for example, are deeply preferred by consumers in certain markets in China, with their selling price being more than twice that of ordinary eggs. Therefore, improving eggshell qualities through reasonable hybridization technology is an effective method to promote the sustainable development of indigenous chicken breeds.

Yaoshan chicken (YS) is a local chicken breed known for its good quality of meat and eggs, as well as its strong adaptability to the environment. It is widely raised in the southwest of China [10,11]. However, compared with specialized strains, the low laying performance, with an average egg production rate of 42% and an average egg weight of 45 g, has always been a significant factor hindering the high-quality development of the industry. To improve the egg production of YS, the Guizhou Institute of Animal Husbandry and Veterinary Medicine has carried out breeding selection for 10 generations, resulting in a prominent increase [12]. However, the results of selection breeding in recent generations showed that the average genetic progress in egg production per generation has been significantly slower than in the early stages of breeding selection. Therefore, advancing germplasm innovation and improving the eggshell quality (including eggshell color, strength, and thickness) have become crucial strategies for improving the economic benefits of the YS industry. Subsequently, the GYR crossbred, which carries the blood of YS and is capable of producing green-shelled eggs, was developed through the hybridization of the green-shelled chicken with yellow plumage (GY), recessive white plumage layers with good eggshell quality (RW), and YS chicken, following the crossbreeding pattern (GY♂ × (YS♂ × RW♀)) [13].

Eggshells are synthesized in the uterus (also known as the shell gland) of laying hens, and this process is the longest step in the procedure of egg formation, lasting up to 18 h [14]. Calcium carbonate is the main component of eggshell, and calcium and bicarbonate ions provided by blood in the uterus through transepithelial transport are the raw materials for its synthesis [15]. Jing Wang et al. revealed that the calcium content in eggshell has a significant influence on the thickness and strength of eggshell (*p* < 0.05) [16]. Therefore, any factors that can influence the levels of calcium and bicarbonate ions in the blood of laying hens play an important role in the formation and quality of eggshells. The last 3–4 h of eggshell formation is an important phase for the development of eggshell color [17]. The amount and proportion of three eggshell pigments (protoporphyrin-IX, biliverdin, and zinc chelate of biliverdin) deposited on the surface of the eggshell are the primary factors contributing to the variation in shell color [18]. The biliverdin and its zinc chelate are the main raw materials for the formation of green eggshells, and the shade of green is determined by the biliverdin/protoporphyrin-IX ratio [19]. Due to the significant difference in eggshell color, the GYR hens, which lay green-shelled eggs, and YS hens, which lay light-brown eggs, serve as ideal models for studying the molecular mechanism of eggshell pigmentation.

Multi-omics strategies play an important role in analyzing traits [20]. Transcriptomics is a discipline that was first developed and is widely used in the study of the transcription and regulation of genes in cells, including single-cell transcriptome [21,22,23]. Metabolomics, on the other hand, is an emerging discipline focused on the qualification and quantification of the changes in metabolites caused by transcriptional regulation [24]. The association analysis between transcriptome and metabolome can systematically reveal the functions and regulatory mechanisms of molecules [25]. Reproductive tract microbes also play an important role in reproductive function [26]. The correlation between the reproductive tract microbiota and speckled eggs in laying hens was detected [27]. To our knowledge, the previous studies on avian eggshell qualities have primarily been limited to the transcriptome level [28,29,30,31,32]. Therefore, in order to explore the molecular mechanism of the difference in eggshell quality, we conducted an integrative analysis of transcriptome and metabolome to investigate the eggshell quality traits such as thickness, strength, and shell color in GYR and YS chickens. Our goal is to provide new insights into the utilization of poultry breeds and the study of eggshell quality.

## 2. Materials and Methods

### 2.1. Experimental Animals and Tissue Collection

**Figure 1:**
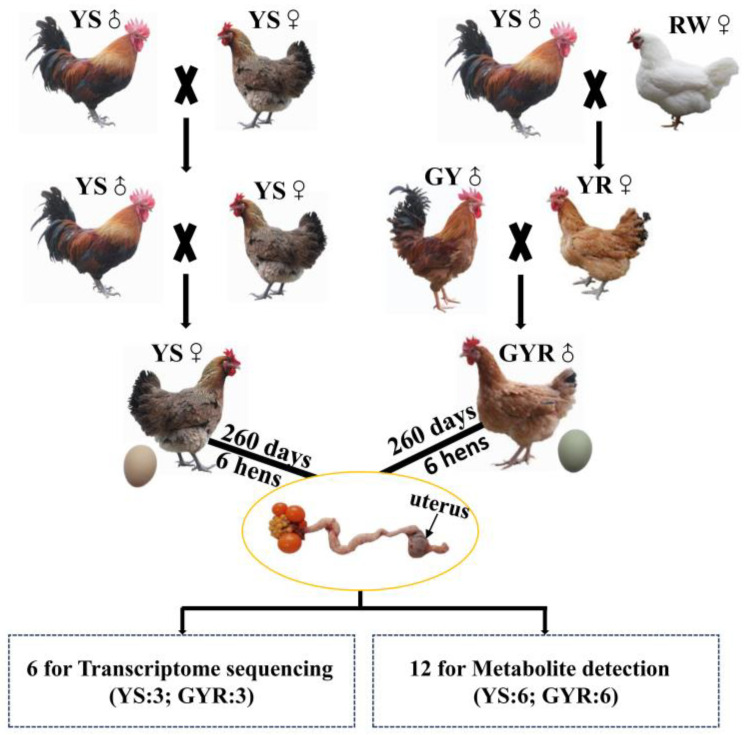
The flowchart of the uterus samples obtained from the experimental chickens for RNA-seq and metabolite identification. YS: Yaoshan chicken; RW: recessive white plumage layers; YR: offspring of YS (♂) and RW (♀); GY: green-shelled chicken with yellow plumage; GYR: offspring of GY(♂) and YR (♀).

The Animal Welfare and Ethics Committee of Guizhou Institute of Animal Husbandry and Veterinary Medicine approved the experimental procedures (AWE-GZSXMSY-2023-07). The experimental chicken flocks in this study were raised at Guiyang Lvyuan Poultry Co., Ltd. (Guiyang, China). A total of 1000 birds were used in this study, consisting of 500 GYR chickens and 500 YS chickens. The GYR was constructed according to the hybridization model of (GY♂ × (YS♂ × RW♀)), where YS was the purebred offspring of Yaoshan chicken. The laying hens were artificially inseminated with roosters at a rate of 1:9. The incubation and feeding conditions of each stage for GYR and YS were consistent. The vaccination program strictly abided by the relevant rules of the local Department of Husbandry and Veterinary. Food and water were freely accessible to the chickens, and the nutrient composition and levels of the feed at different stages are shown in Table 1. During the chick-rearing period (0–6 weeks of age, Phase I), the stocking density was 50 birds/m^2^, the environmental temperature gradually decreased from 32 °C to 23 °C, and the lighting duration reduced from 24 h to 9 h per day. During the grower period (7–18 weeks of age, Phase II), the stocking density was reduced to 12 birds/m^2^, and the environmental temperature and lighting duration were maintained at 23 °C and 9 h/day, respectively. During the laying period (19–37 weeks of age, Phase III), the hens were housed individually in single cages. The lighting duration increased from 9 h to 16 h per day and remained stable after 25 weeks of age. The housing temperature was maintained at 23 °C, and the health status and eggshell color of each chicken were monitored. Sick chickens and those showing abnormal behavior due to human interference were removed. At 260 days of age, eggshell qualities (color, thickness, and strength) were measured, with 30 eggs chosen randomly from each group [1,33]. The eggshell color and thickness were measured by averaging values from three locations on the egg (the sharp end, equator, and blunt end). A colorimeter (Shenzhen Threenh Technology Co., Ltd., Shenzhen, China) was used to measure eggshell color, while a vernier caliper (Ningbo Deli Tools Co., Ltd., Ningbo, China) was used to measure eggshell thickness. Additionally, eggshell strength at the blunt end was evaluated using an eggshell force tester (Tenovo International Co., Ltd., Beijing, China). A total of 12 healthy laying hens were humanely euthanized during the period of eggshell calcification, six from the GYR group and six from the YS group. The uterine tissues of chicken were collected and quickly frozen in liquid nitrogen and then stored in a refrigerator at −80 °C for subsequent RNA extraction. The detailed flowchart of the production of GYR and YS chickens, along with the uterine tissue sampling procedure, is shown in Figure 1.

### 2.2. Transcriptome Sequencing and Data Analysis

The total RNA of GYR and YS’s uterine tissues was isolated using TRIzol RNA extraction reagent (Tiangen Biotech Co., Ltd., Beijing, China). Six high-quality RNA samples (three samples from each group) were transported to Metware Biotechnology Co., Ltd. (Wuhan, China) for 150bp paired-end sequencing on an Illumina NoveSeq 6000 Platform. The average sequencing depth was 6×, and the bases over 42.20 G for six samples were acquired. Fastp (version 0.23.4) was applied to evaluate and filter the raw data [34]. Reads containing more than 10% unknown bases or more than 50% low-quality bases (Q ≤ 20) were removed. The high-quality data, comprising 41.15 G bases, had their Q20, Q30, and GC content calculated from the above processes. These clean data were used for all subsequent analyses. The reference genome (GRCg7b.110) and its annotation files were obtained from the Ensembl database (https://www.ensembl.org/Gallus_gallus/Info/Index, accessed on 10 November 2023). Indexes of the reference genome were constructed through HISAT2 (version 2.2.1), and the clean data were aligned to them, generating output files in SAM format [35]. SAMtools (version 1.1.4) was used to transform the SAM format of files into BAM format and building indexes, which would be utilized for quantification analysis [36]. The genome annotation and alignment files were used to calculate the mapped reads for each gene in the samples using featureCouts (version 2.0.6), which provided raw counts for differential expression analysis (DEGs) [37]. DEGs between GYR and YS were detected utilizing the edgeR package (version 3.40.2) in R (version 4.2.2) with the screening standards of |log_2_ (fold change)| > 1 and *p*-value < 0.05, which would be used to plot a volcano map and correlation heatmap for visualization [38]. The R packages clusterProfiler (version 4.6.2) and org.Gg.eg.db (version 3.16.0) were used to perform functional enrichment analysis of genes, including Gene Ontology (GO) and *Kyoto Encyclopedia of Genes and Genomes (KEGG)* [39].

### 2.3. Metabolites Detection and Analysis

All 12 uterus samples (six for each group) were packed in foam boxes containing dry ice and sent to Metware Biotechnology Company (Wuhan, China) for metabolites determination, which was based on the liquid chromatography–tandem mass spectrometry (LC-MS/MS). A quality control (QC) sample prepared by mixing equal amounts of all tested samples was used to evaluate the accuracy and reliability of the detection results [40]. The identification of all metabolites was performed using the self-established target standard database MWDB (containing secondary spectra and retention time (RT)), the public MHK database (including information from KEGG, HMDB, and Metlin, along with secondary spectra and RT), provided by Metware Biotechnology Co., Ltd., and the online software MetDNA2 (version 1.4.1, http://metdna.zhulab.cn/metdna/, accessed on 8 July 2024). The quantification of metabolites was performed utilizing the multiple reaction monitoring (MRM) of triple quadrupole mass spectrometry. The parent ions of the aimed compounds eventually changed into typical characteristic fragment ions after experiencing screening, collision-induced ionization, and filtration. Analyst (version 1.6.3) and MultiaQuant (version 3.0.2) were used to integrate and correct the detected chromatographic peaks whose areas indicated the relative content of the corresponding compounds [41]. Orthogonal partial least squares discriminant analysis (OPLS-DA) was utilized to analyze the variability between different groups and within group samples [42]. To identify differential metabolites, the variable importance in projection (VIP) obtained from the OPLS-DA model and fold change were used jointly. The |log_2_ (fold change)| > 1 and VIP > 1 were the screening criteria. Functional annotation and enrichment analysis of differential metabolites (DMs) were performed using the KEGG database, and the relevant graphics used to present the results of metabolites analysis were generated using R (version 4.2.2) [43].

### 2.4. Integrated Analysis of Transcriptome and Metabolome

Based on the previously identified differentially expressed genes (DEGs) and differential metabolites (DMs), a Pearson correlation test was performed to evaluate the relationship between DEGs and DMs. A nine-quadrant graph was generated to visualize the trends in their changes [44]. Additionally, to further explore the interactions between DEGs and DMs, the MetaboAnalystR package (version 6.0) was utilized to construct an interaction network diagram [45].

### 2.5. Statistical Analysis

Microsoft Excel (version 2021) was used to organize all measured data on eggshell qualities (color, thickness, and strength). When the data in each group followed a normal distribution (Shapiro-Wilk’s test, *p*-value ≥ 0.05) and the variance between groups was homogeneous (Levene’s test, *p*-value ≥ 0.05), an independent sample *t*-test was conducted using IBM SPSS Statistics (version 19) to assess the differences between GYR and YS. Otherwise, the Mann–Whitney U test would be applied. A significance level of *p*-value < 0.05 was considered statistically significant, while *p*-value < 0.01 was considered highly significant, and the results were expressed as “Mean ± SEM”. Additionally, Spearman’s rank correlation analysis was conducted using R (version R 4.2.5) to assess the relationships between variables. The correlation coefficient closer to 1 or −1, with a *p*-value less than 0.05, indicates a strong positive or negative correlation, respectively.

## 3. Results

### 3.1. Differences in Eggshell Quality Between the YS and GYR

**Figure 2:**
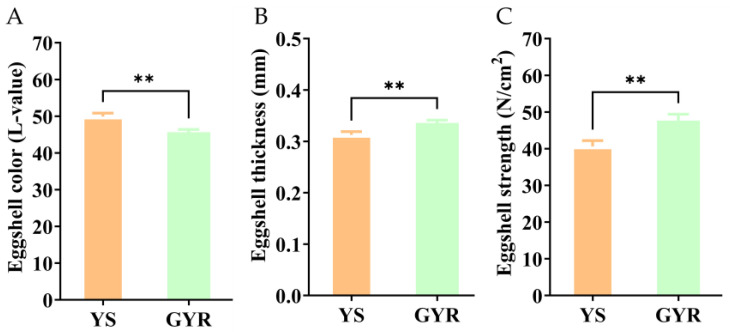
The differences in eggshell qualities between YS and GYR: (**A**) the difference in L-value of eggshell color between YS and GYR; (**B**) the difference in thickness of eggshell between YS and GYR; and (**C**) the difference in strength of eggshell between YS and GYR. All data in treatments were presented as a Mean value and SEM; ** above the error bars indicate extremely significant differences between experimental groups (*p* < 0.01).

Through standardized care for 37 weeks, at 260 days of age, 30 eggs separately produced by GYR and YS were randomly selected to measure eggshell quality. The differences in color, thickness, and strength of eggshells for GYR and YS are shown in Figure 2 and Table S1. The eggs produced by GYR had green shells, while the YS eggs had light-brown shells, resulting in a significant difference in the L-value of eggshell color (Figure 2A). In addition, GYR eggs had greater thickness (Figure 2B) and strength (Figure 2C) in eggshells compared with YS eggs. These results indicate that the eggshell quality of GYR is better than that of YS.

### 3.2. DEGs Analysis Between YS and GYR

**Figure 3:**
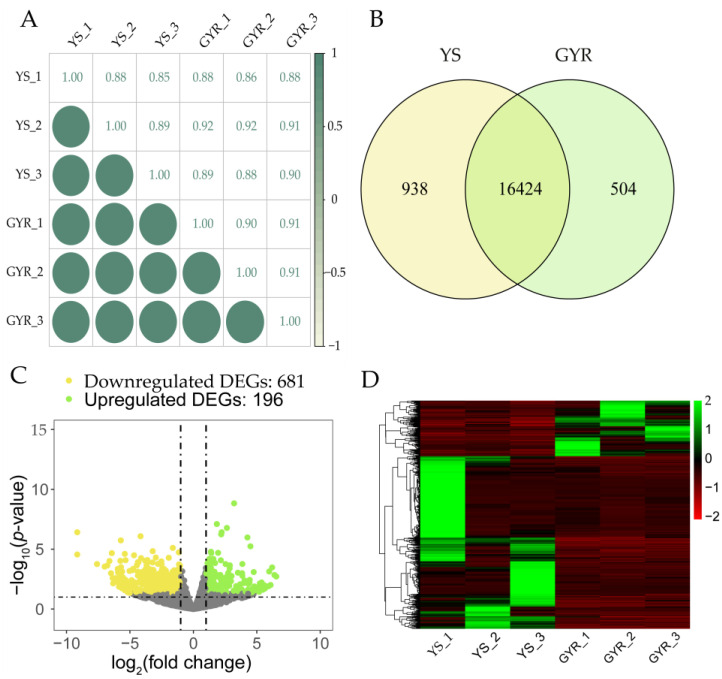
The analysis of DEGs between YS and GYR: (**A**) Spearman correlation analysis of genes between samples; (**B**) Venn diagram of gene expression in YS and GYR; and (**C**) volcano plot of DEGs between YS and GYR; and (**D**) heatmap of DEGs between YS and GYR.

After quality control, a total of 276,912,138 clean reads were obtained from the RNA-seq data for the uterine tissues of YS and GYR chickens. The average number of reads for the YS group was 46,801,101.3, with about 90.27% of each sample successfully mapped to the reference genome (GRCg7b.110). For the GYR group, the average number of reads was 45,502,944.7, with about 90.7% of each sample successfully mapped to the reference genome (GRCg7b.110). The Q30 of all samples was greater than 91%, and the GC content ranged from 51.42% to 51.97% (Table 2). In addition, the correlation coefficients of gene expression levels between samples were greater than 0.85 (Figure 3A), which demonstrates that the selection of experimental chickens and the RNA sequencing data are reliable. As shown in Figure 3B, a total of 17,866 genes were expressed in the uterine tissues of YS and GYR, of which 16,424 were shared by both 938 and 504 genes expressed uniquely in YS and GYR, respectively. The differential expression analysis of genes between YS and GYR revealed a total of 877 differentially expressed genes (DEGs), including 196 upregulated genes and 681 downregulated genes in the GYR group (Figure 3C, Table S2). Cluster analysis of the DEGs showed that samples from the same group clustered together, while there was a significant difference in gene expression between the YS and GYR groups (Figure 3D). The detailed information for the top 15 upregulated DEGs (*SLCO1B3*, *SLCO1B1*, *PTGR1*, *LGR6*, *MELTF*, *PSAT1*, *AVD*, *AGBL3*, *CRMP1*, *LOC419409*, *ZFPM1*, *GALC*, *ATP6V0A4*, *SLC28A3*, and *USP43*) and the downregulated DEGs (*CRISP2*, *GVINP1*, *LOC112532804*, *OVSTL*, *LOC121106625*, *LOC100857858*, *CCDC141*, *CCL19*, *CSMD1*, *LOC100857191*, *LOC121106469*, *SCIN*, *RHOBTB1*, *C10H15ORF48*, and *LOC121112483*) between YS and GYR are presented in Table 3.

### 3.3. Functional Enrichment Analysis of DEGs

**Figure 4:**
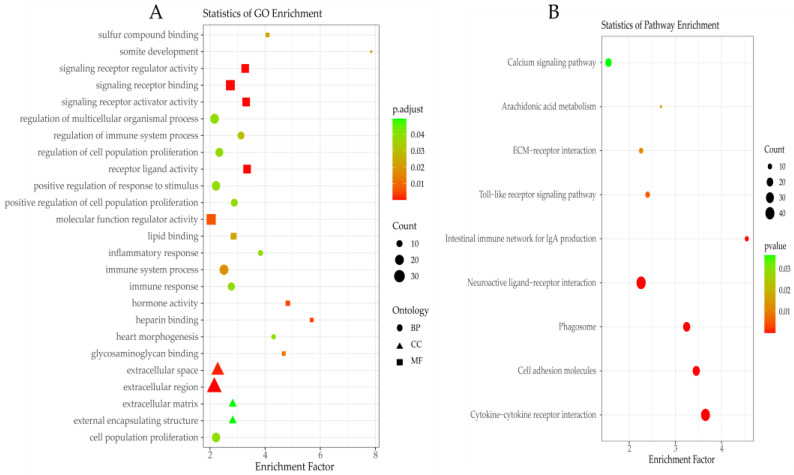
The enrichment analysis of DEGs: (**A**) GO analysis of DEGs and (**B**) KEGG analysis of DEGs.

GO and KEGG enrichment analysis were utilized to classify the function of DEGs. A total of 25 significantly enriched GO terms were detected in (Figure 4A, Table S3), including biological processes (immune system process, somite development, regulation of immune system process, immune response, positive regulation of cell population proliferation, regulation of multicellular organismal process, heart morphogenesis, inflammatory response, cell population proliferation, positive regulation of response to stimulus, and regulation of cell population proliferation), cellular components (extracellular region, extracellular space, external encapsulating structure, and extracellular matrix) and molecular functions (signaling receptor binding, receptor–ligand activity, signaling receptor activator activity, signaling receptor regulator activity, heparin binding, hormone activity, molecular function regulator activity, glycosaminoglycan binding, sulfur compound binding, and lipid binding). The significantly enriched pathways of KEGG for DEGs are shown in Figure 4B and Table S4, including cytokine–cytokine receptor interaction, cell adhesion molecules, phagosome, neuroactive ligand–receptor interaction, intestinal immune network for IgA production, toll-like receptor signaling pathway, ECM–receptor interaction, arachidonic acid metabolism, and calcium signaling pathway.

### 3.4. DMs Analysis Between YS and GYR

**Figure 5:**
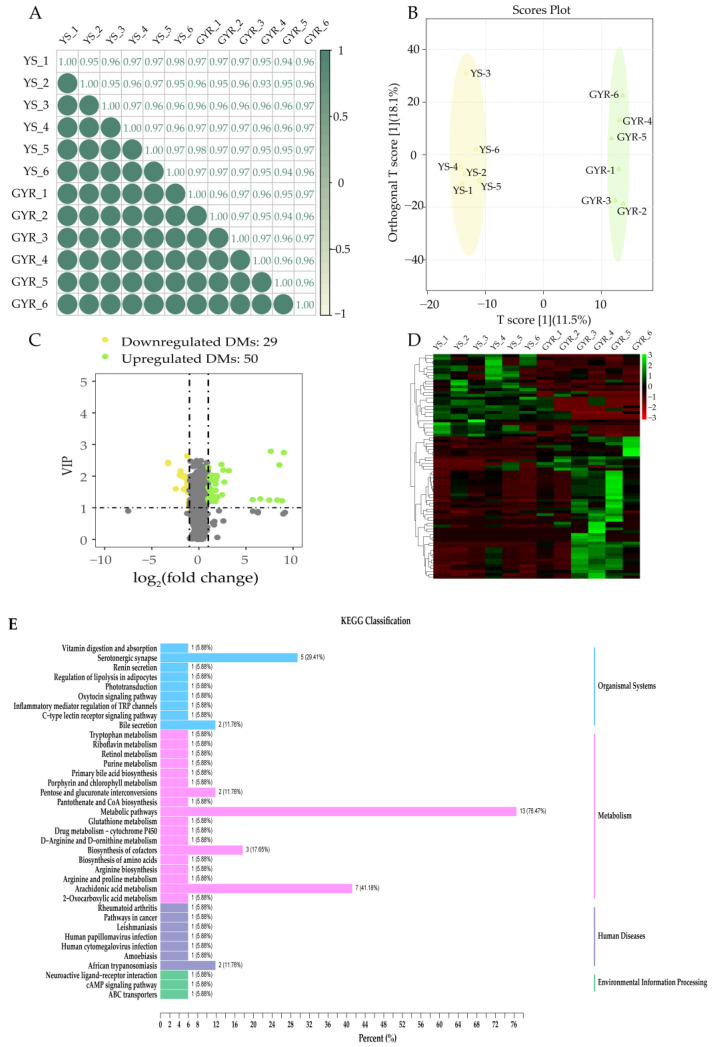
The analysis of DMs between YS and GYR: (**A**) Spearman correlation analysis of metabolites between samples; (**B**) OPLS-DA scores plot for YS and GYR; (**C**) volcano plot of DMs between YS and GYR; (**D**) heatmap of DMs between YS and GYR; and (**E**) KEGG classification of DMs between YS and GYR. The correlation coefficient, closer to 1 or −1, with a *p*-value < 0.05, indicates a strong positive or negative correlation, respectively.

In this study, a total of 1619 metabolites were identified in uterus samples of YS and GYR chickens (six for each group). The Spearman correlation analysis of metabolite contents indicated high biological reproducibility among samples, with correlation coefficients exceeding 0.9, satisfying the experimental requirements (Figure 5A). Orthogonal partial least squares discriminant analysis (OPLS-DA) for all metabolites revealed minimal differences within the same group, while a significant distinction was observed between the YS and GYR groups (Figure 5B). In addition, 79 significantly differential metabolites (DMs) were identified using the thresholds |log_2_ (fold change)| > 1 and VIP > 1. Of these, 50 DMs were upregulated, while 29 DMs were downregulated in the GYR group (Figure 5C, Table S5). Cluster analysis of the 79 DMs showed that samples from the same group were closely clustered, while a clear distinction was observed between the YS and GYR groups (Figure 5D); the detailed information for the 79 DMs is shown in Table S5. The KEGG enrichment analysis showed that all the DMs were enriched in 37 pathways, including 18 related to metabolism, 9 related to organismal systems, 7 related to human diseases, and 3 related to environmental information processing (Figure 5E, Table S6).

### 3.5. Integrated Analysis of Transcriptome and Metabolome

**Figure 6:**
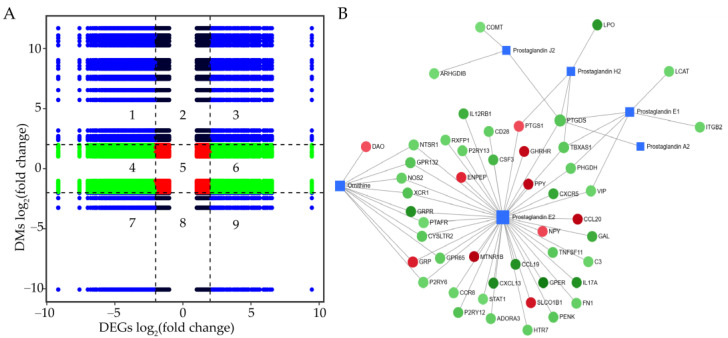
The correlation analysis between DEGs and DMs: (**A**) nine-quadrant diagram of correlation between DEGs and DMs; and (**B**) interaction network diagram between DEGs and DMs. The circles represent DEGs, while the squares represent DMs. The red indicates upregulation, whereas blue signifies downregulation.

A nine-quadrant diagram was used to illustrate the correlation between all DEGs and DMs obtained through comparative transcriptomic and metabolomic analyses (Figure 6A, Table S7). The DEGs and DMs located in quadrants 3 and 7 exhibited a positive correlation, whereas those in quadrants 1 and 9 displayed a negative correlation, suggesting that changes in metabolite accumulation within these quadrants may be regulated either positively or negatively by corresponding genes. Additionally, 48 DEGs and 6 DMs were mapped to the interaction network diagram of DEGs and DMs generated by MetaboAnalystR (Figure 6B, Table S8), including 10 upregulated and 38 downregulated genes, with prostaglandin E2 showing the highest degree and betweenness.

## 4. Discussion

Eggshell quality varies with breed and is influenced by factors such as genetics, nutrition, environment, and age [46]. Although there is no direct correlation between eggshell quality and the nutrient content of eggs, poor eggshell quality can negatively impact the interests of layer poultry enterprises by affecting the consumers’ preference and increasing the breakage ratio before sale [47]. In general, the eggshell quality of local chicken breeds is inferior to that of commercial breeds [48]. Therefore, improving the eggshell quality of local chicken breeds has always been a primary concern for egg producers and breeders. In this study, two commercial breeds of layers with superior eggshell quality (recessive white-feathered chickens laying eggs with thicker and stronger eggshells and yellow-feathered chickens laying green-shelled eggs) were used to improve the eggshell quality of YS, an indigenous breed in China. The eggshell quality traits of YS and its hybrids (GYR) were measured, and the result showed that GYR exhibited better genetic advantages in the color, thickness, and strength of eggshells (Figure 1). The formation process of the eggshell is highly complex and meticulously regulated by genetic and biological pathways within the uterus, which serves as a pivotal tissue for investigating eggshell quality traits [49]. In this study, transcriptomic and metabolomic analyses of uterine tissues were employed to explore the underlying molecular mechanisms responsible for the observed differences in eggshell quality between YS and GYR chickens. The findings provide theoretical support for the hybrid improvement of YS chickens and offer novel insights for future research on eggshell quality in poultry.

In this study, a total of 877 DEGs in uteruses between YS and GYR were identified, with 196 upregulated genes and 681 downregulated genes in GYR compared to YS. These results indicate that these DEGs may be critical in the regulation of eggshell quality. The top five upregulated DEGs were *SLCO1B3* (solute carrier organic anion transporter family member 1B3), *SLCO1B1* (solute carrier organic anion transporter family member 1B1), *PTGR1* (prostaglandin reductase 1), *LGR6* (leucine-rich repeat-containing G protein-coupled receptor 6), and *MELTF* (melanotransferrin), whereas the top five downregulated DEGs were *CRISP2* (cysteine-rich secretory protein 2), *GVINP1* (GTPase, very large interferon inducible pseudogene 1), *LOC112532804* (uncharacterized LOC112532804), *OVSTL* (ovostatin-like), and *LOC121106625* (guanylate-binding protein 1-like). *SLCO1B3* is a protein-coding gene with a retroviral (EAV-HP) insertion in its 5’ flanking region, and it plays a role in the transport of bile salts. High expression of this gene in the shell gland can lead to the formation of green-shelled eggs [50]. *SLCO1B1*, located 26 kb downstream of the EAV-HP insertion site, is adjacent to *SLCO1B3*. As a member of the solute carrier organic anion transporter family, *SLCO1B1* plays a critical role in the regulation of eggshell pigmentation, particularly influencing the development of eggshell brownness [51]. *PTGR1*, a critical regulator of prostaglandin synthesis, catalyzes the metabolism of eicosanoids such as PGE2 and lipoxin A4, thereby playing a central role in modulating inflammatory and antioxidant responses [52]. *LGR6* is capable of modulating and enhancing canonical Wnt (cWNT) signaling via R-Spondin (RSPO1–4) in various stem cell environments across the body [53]. *MELTF* plays a regulatory role in ferroptosis, pyroptosis, and autophagy during the process of endometrial epithelium regeneration mediated by platelet-rich plasma (PRP) [54]. *CRISP2* modulates sperm function and male fertility by influencing the acrosome reaction and contributing to the establishment of a normal sperm flagella waveform [55]. *GVINP1* selectively and non-covalently binds to guanosine triphosphate (GTP) and has been identified as an independent prognostic marker for patients with lung adenocarcinoma (LUAD) and non-small cell lung cancer (NSCLC) [56]. The ovostatin-like protein encoded by *OVSTL*, located in the cuticle layer of eggshells, exhibits antibacterial properties and may play a role in molecular adhesion and resistance to microbial invasion [57]. The results of the enrichment analysis revealed that the DEGs were significantly enriched in 25 GO terms and 9 KEGG pathways, which may play an important role in regulating the eggshell quality. Notably, several immune-related pathways in the GO terms, including immune system process, regulation of immune system process, and immune response, were identified. These pathways may contribute to protecting chickens from bacterial infections by enhancing the immune response [58]. Subsequently, the calcium signaling pathway, identified through KEGG pathway enrichment analysis, was also observed in the uterine tissues of Rhode Island White hens with different eggshell qualities (eggshell thickness and strength) [59]. Furthermore, the neuroactive ligand–receptor interaction pathway was found to be associated with eggshell glossiness [60], and the arachidonic acid metabolism pathway was related to the improvement of eggshell quality in eggs from induced molting chickens [61]. These findings further suggest that these pathways may play a crucial role in the regulation of eggshell quality.

In this study, a total of 1619 metabolites were identified, with 79 differential metabolites (DMs) detected between the two groups. Among these, 50 DMs were upregulated, while 29 DMs were downregulated in GYR compared to YS. There were 14 prostaglandin-related DMs (11β-13,14-dihydro-15-keto prostaglandin F2α, prostaglandin A2, PGD1, PGE1, prostaglandin B2, 13,14-dihydro-15-keto PGD2, PGJ2, 13,14-dihydro-15-keto PGE2, prostaglandin H2, 11β-prostaglandin E2, prostaglandin E2, PGF2α, PGK1, and 1a,1b-dihomo PGF2). Prostaglandins exert multiple regulatory effects during the formation of poultry eggshells, primarily by regulating calcium metabolism, uterine contractions, and collagen synthesis, eventually influencing the formation and hardness of the eggshell [62,63,64]. Additionally, studies have demonstrated that biliverdin plays a key role in the formation of green-shelled eggs in poultry [65,66]. The biliverdin detected in this study was found to be significantly upregulated in GYR chickens producing green-shelled eggs, which aligns with the findings of previous research. KEGG enrichment analysis of DMs identified a total of 37 pathways, including the neuroactive ligand–receptor interaction and arachidonic acid metabolism pathways. Importantly, these pathways were also identified in the KEGG enrichment analysis of DEGs, suggesting a close association between these pathways and eggshell quality. Furthermore, two signaling pathways (bile secretion and primary bile acid biosynthesis) related to bile synthesis and secretion were detected. The expression of *SLCO1B3* in the shell gland can cause bile salt to enter the shell gland, where biliverdin is secreted into the eggshell and forming a blue shell. The expression of *SLCO1B3* in the shell gland facilitates the entry of bile salts into the gland, where biliverdin is subsequently secreted into the eggshell, contributing to the formation of a green eggshell [67]. Therefore, signaling pathways involved in bile synthesis and secretion are key factors influencing the coloration of green eggshells.

The correlation analysis between DEGs and DMs revealed that 48 DEGs and 6 DMs were mapped to the interaction network. Among these, five DMs (prostaglandin E2, prostaglandin J2, prostaglandin H2, prostaglandin E1, and prostaglandin A2) were related to prostaglandins. Notably, prostaglandin E2 and *PTGDS* exhibited the highest degree of betweenness in DMs and DEGs, respectively. An increase in serum prostaglandin E2 levels can influence calcium deposition in eggshells, thereby enhancing eggshell quality [64]. *PTGDS* is a protein-coding gene, and its encoded enzyme (prostaglandin D2 synthase) can catalyze the conversion of prostaglandin H2 to prostaglandin D2 [68]. What is more, prostaglandin H2 is also a precursor for the synthesis of prostaglandin E2 [69]. Therefore, *PTGDS* can indirectly influence the synthesis rate of prostaglandin E2, thereby impacting eggshell quality. In this study, the expression of the *PTGDS* gene in the uteruses of GYR chickens was significantly downregulated, leading to reduced synthesis of prostaglandin D2 synthase. We speculate that this reduction in prostaglandin D2 synthesis may result in decreased utilization of prostaglandin H2, thereby increasing the production of prostaglandin E2, which could contribute to improved eggshell quality (eggshell thickness and strength). In addition, the upregulated gene with the highest degree and betweenness was *PTGS1*, which encoded COX1, a key enzyme during prostaglandin biosynthesis. COX1 can catalyze the conversion of arachidonic acid into prostaglandin H2 and ultimately lead to an increase in prostaglandin E2 [70]. Overall, the gene–metabolite pairs *PTGDS–*prostaglandin E2 and *PTGS1–*prostaglandin E2 are the most crucial regulators of eggshell thickness and strength. These pairs play a pivotal role in the modulation of eggshell characteristics, highlighting their importance in the genetic and metabolic pathways underlying eggshell formation.

## 5. Conclusions

In conclusion, this study leveraged transcriptomic and metabolomic to study the uterine tissues of YS and GYR chickens with different eggshell qualities. We identified genes potentially involved in the regulation of eggshell traits, including *SLCO1B3*, *SLCO1B1*, *PTGR1*, *LGR6*, *MELTF*, *CRISP2*, *GVINP1*, and *OVSTL*, et al., as well as key uterine metabolites such as prostaglandin-related DMs and biliverdin. Additionally, we identified several critical pathways, such as calcium signaling, neuroactive ligand–receptor interaction, arachidonic acid metabolism, bile secretion, and primary bile acid biosynthesis, which may contribute to the observed differences in eggshell quality between the two chicken breeds. Furthermore, through association analysis between DEGs and DAMs, we identified two significant gene–metabolite pairs: *PTGDS–*prostaglandin E2 and *PTGS1–*prostaglandin E2. These findings provide a deeper understanding of the molecular mechanisms regulating eggshell quality and offer valuable insights for the hybrid improvement of YS chicken. Overall, this study provides new avenues for exploring the metabolic and genetic interactions that influence eggshell traits and lays a theoretical foundation for studying the molecular mechanisms underlying eggshell quality in other poultry breeds, thereby contributing to the production of high-quality eggs.

## Figures and Tables

**Table 1:** Ingredients and nutrient levels of the basal diets for experimental chicken.

Items	Phase I ^1^ (%)	Phase II ^1^ (%)	Phase III ^1^ (%)
Ingredients			
Maize	65.0	64.0	60.0
Soybean meal	31.0	25.0	28.0
Soybean oil	–	–	1
Wheat bran	–	7.0	–
Limestone	–	–	7
Premix ^2^	4.0	4.0	4.0
Total	100	100	100
Nutrient levels			
ME/(MJ/kg)	11.92	11.30	11.51
Crude protein/%	18.0	14.0	16.5
Crude fat/%	3.0	3.0	3.0
Crude fiber/%	3.0	3.0	3.0
Calcium/%	0.90	1.00	3.20
Available P/%	0.45	0.40	0.40
NaCl/%	0.36	0.36	0.32
Met+Cys/% Lys/%	0.71 0.85	0.56 0.55	0.64 0.69

^1^ Phase I is 0–6 weeks of age; Phase II is 7–18 weeks of age; Phase III is 19–37 weeks of age; ^2^ the premix per kilogram provided Vitamin A 11,000 IU; Vitamin D3 3800 IU; Vitamin E 16 IU; Vitamin K 3 mg; Vitamin B12 2 mg; thiamine 1.5 mg; riboflavin 4 mg; calcium pantothenate 5 mg; niacin 20 mg; pyridoxine 5 mg; biotin 2 mg; folate 3 mg; choline 550 mg; Fe 58 mg; Mn 60 mg; Zn 75 mg; Se 0.4 mg; Cu 7 mg; and I 1 mg.

**Table 2:** Summary of quality assessment and mapping for RNA-seq data.

Sample	Raw Reads	Clean Reads	Mapping (%)	Q20 (%)	Q30 (%)	GC (%)
YS_1	48,666,990	47,833,650	88.8	96.98	91.94	51.97
YS_2	49,079,746	48,281,330	91.0	97.16	92.32	51.10
YS_3	44,958,804	44,288,324	91.0	96.92	91.71	51.77
GYR_1	45,735,116	45,072,044	90.9	97.18	92.36	51.79
GYR_2	45,695,862	45,033,400	91.0	97.13	92.18	51.42
GYR_3	47,173,102	46,403,390	90.2	97.26	92.62	51.59

**Table 3:** Top 15 DEGs with upregulation and downregulation between YS and GYR, with *p*-value and adjusted *p*-value.

Gene	log_2_ (Fold Change)	*p*-Value	*p*-Adjust	Regulated
*SLCO1B3*	9.4677	1.41 × 10^−24^	3.17 × 10^−20^	up
*SLCO1B1*	3.2076	1.48 × 10^−09^	1.67 × 10^−05^	up
*PTGR1*	1.8471	7.93 × 10^−08^	6.00 × 10^−04^	up
*LGR6*	2.6386	1.68 × 10^−07^	9.00 × 10^−04^	up
*MELTF*	2.2275	3.87 × 10^−07^	1.50 × 10^−03^	up
*PSAT1*	2.1938	5.79 × 10^−07^	1.90 × 10^−03^	up
*AVD*	4.2607	1.05 × 10^−06^	2.60 × 10^−03^	up
*AGBL3*	4.4962	5.75 × 10^−06^	1.08 × 10^−02^	up
*CRMP1*	1.3833	1.64 × 10^−05^	1.69 × 10^−02^	up
*LOC419409*	1.9462	2.01 × 10^−05^	1.86 × 10^−02^	up
*ZFPM1*	1.4061	2.09 × 10^−05^	1.86 × 10^−02^	up
*GALC*	1.2642	6.43 × 10^−05^	4.11 × 10^−02^	up
*ATP6V0A4*	1.5728	6.50 × 10^−05^	4.11 × 10^−02^	up
*SLC28A3*	2.1652	8.48 × 10^−05^	4.60 × 10^−02^	up
*USP43*	1.3754	1.00 × 10^−04^	5.84 × 10^−02^	up
*CRISP2*	−9.1557	3.86 × 10^−07^	1.50 × 10^−03^	down
*GVINP1*	−4.1667	8.31 × 10^−07^	2.30 × 10^−03^	down
*LOC112532804*	−5.7186	1.85 × 10^−06^	4.10 × 10^−03^	down
*OVSTL*	−1.6217	7.81 × 10^−06^	1.20 × 10^−02^	down
*LOC121106625*	−3.2059	1.47 × 10^−05^	1.59 × 10^−02^	down
*LOC100857858*	−1.1501	1.84 × 10^−05^	1.80 × 10^−02^	down
*CCDC141*	−6.4296	2.14 × 10^−05^	1.86 × 10^−02^	down
*CCL19*	−5.4059	2.46 × 10^−05^	2.06 × 10^−02^	down
*CSMD1*	−9.1551	2.79 × 10^−05^	2.23 × 10^−02^	down
*LOC100857191*	−4.6780	3.23 × 10^−05^	2.43 × 10^−02^	down
*LOC121106469*	−2.9620	4.98 × 10^−05^	3.63 × 10^−02^	down
*SCIN*	−1.9398	5.23 × 10^−05^	3.69 × 10^−02^	down
*RHOBTB1*	−4.1245	5.59 × 10^−05^	3.83 × 10^−02^	down
*C10H15ORF48*	−3.6282	8.13 × 10^−05^	4.60 × 10^−02^	down
*LOC121112483*	−5.3421	8.36 × 10^−05^	4.60 × 10^−02^	down

## Data Availability

The original data presented in this study are openly available in the NCBI (https://www.ncbi.nlm.nih.gov/) under Bioproject accession number PRJNA1211182.

## Supplementary Materials

The following supporting information can be downloaded at https://www.mdpi.com/article/10.3390/genes16040383/s1, Table S1: Eggshell qualities of YS and GYR chickens; Table S2: All DEGs between YS and GYR; Table S3: GO terms of DEGs between GYR and YS; Table S4: KEGG pathways of DEGs between GYR and YS; Table S5: All DMs between YS and GYR; Table S6: KEGG pathways of DMs between GYR and YS; Table S7: Pearson correlation coefficients of DEGs and DMs; Table S8: Nodes between DEGs and DMs.

## Abbreviations

The following abbreviations are used in this manuscript:

YS	Yaoshan chicken
RW	recessive white plumage layers
YR	offspring of YS (♂) and RW (♀)
LD	linear dichroism
GY	green-shelled chicken with yellow plumage
GYR	offspring of GY(♂) and YR (♀)
DEGs	differentially expressed genes
DMs	differential metabolites
